# Supplementary material for: Observation of giant room-temperature anisotropic magnetoresistance in the topological insulator β-Ag2Te
Source: Nat Commun. 2024 Feb 10;15:1259. doi: 10.1038/s41467-024-45643-y (PMC10858948; doi:10.1038/s41467-024-45643-y)
Supplement: Supplementary file 1 — Supplementary Information [file 41467_2024_45643_MOESM1_ESM.pdf]

Supplementary Materials for

Observation of giant room-temperature anisotropic magnetoresistance in the topological insulator *β*-Ag_2Te

Wei Ai[#, 1], Fuyang Chen[#, 2], Zhaochao Liu[1], Xixi Yuan[3], Lei Zhang[1], Yuyu He[1], Xinyue Dong[1], Huixia Fu[*,3], Feng Luo[*, 1], Mingxun Deng[*, 2], Ruiqiang Wang[2], Jinxiong Wu[*,1]

[1]*Tianjin Key Lab for Rare Earth Materials and Applications, Center for Rare Earth and Inorganic Functional Materials, Smart Sensor Interdisciplinary Science Center, School of Materials Science and Engineering, Nankai University, Tianjin 300350, China*

[2]*Guangdong Provincial Key Laboratory of Quantum Engineering and Quantum Materials, School of Physics and Telecommunication Engineering, South China Normal University, Guangzhou 510006, China*

[3]*Center of Quantum Materials and Devices & College of Physics, Chongqing University, Chongqing 401331, China*

[#]*These authors contributed equally to this work.*

**Corresponding author. Email: jxwu@nankai.edu.cn; dengmingxun@scnu.edu.cn; feng.luo@nankai.edu.cn; hxfu@cqu.edu.cn;*

Supplementary Note 1: CVD growth and characterization of *β*-Ag_2Te nanosheets

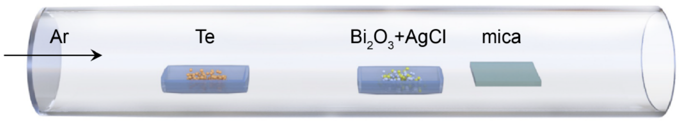

Supplementary Fig. 1 | Schematic illustration of CVD setup to synthesize *β*-Ag_2Te nanosheets on mica substrate. The Te lumps and mixed powders of AgCl and Bi_2O_3 were separately located in two heating zones to act as the evaporation sources.

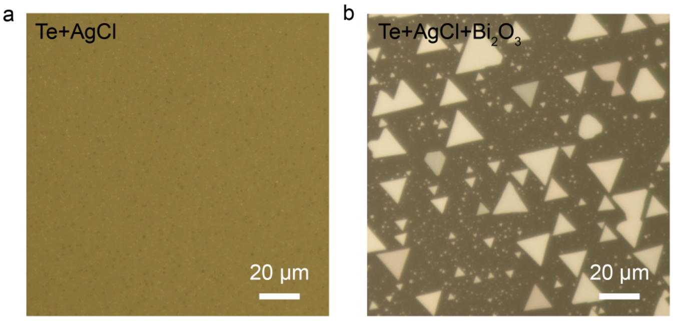

Supplementary Fig. 2 | Comparison experiments conducted to investigate the role of Bi_2O_3 powders on synthesizing *β*-Ag_2Te nanosheets. All the synthetic conditions were kept the same expect for the one of Bi_2O_3. **(a)** without Bi_2O_3, **(b)** with Bi_2O_3 powders. Obviously, merely no nanoplates were obtained on mica substrate if no Bi_2O_3 powders were used under the same CVD condition.

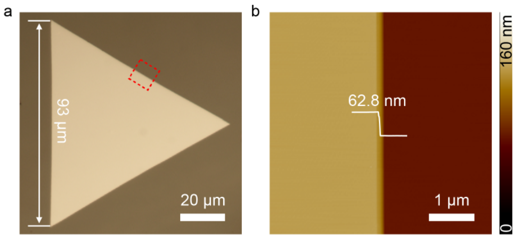

Supplementary Fig. 3 | A CVD-grown *β*-Ag_2Te nanosheet with a large domain size. **(a)** OM image of a CVD-grown *β*-Ag_2Te large single crystal with a domain size of ~93 µm. **(b)** The corresponding AFM image and height profile of red dashed rectangular area in **(a)**, showing a thickness of ~62.8 nm.

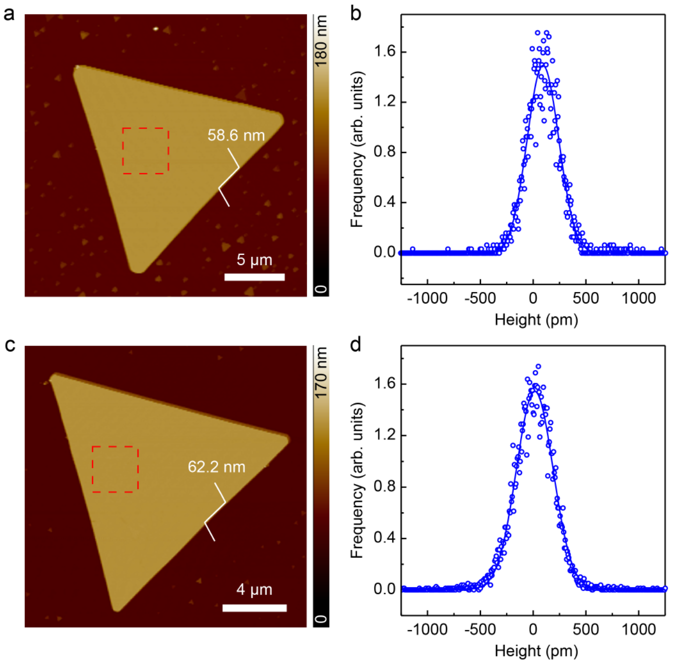

Supplementary Fig. 4 | AFM measurements conducted on typical CVD-grown β-Ag_2Te crystals to evaluate their thicknesses and surface roughness. (a, b) The AFM image and corresponding surface height analysis of a 58.6-nm-thick β-Ag_2Te nanosheet, showing an ultrasmall surface roughness of ~0.14 nm. **(c, d)** The AFM image and corresponding surface height analysis of a 62.2-nm-thick β-Ag_2Te nanosheet.

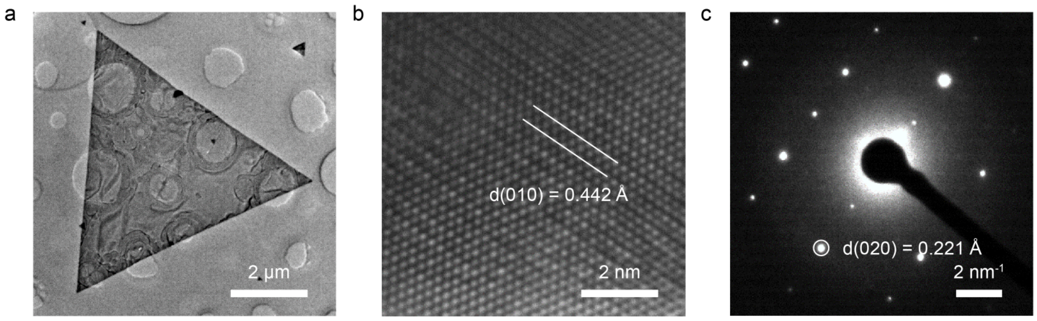

Supplementary Fig. 5 | The *ab*-plane TEM imaging of a CVD-grown β-Ag_2Te nanoplate transferred onto the Cu grid. (a) Low-magnification TEM of the nanoplate with a triangular morphology. **(b)** High-resolution TEM and **(c)** the corresponding selected area electron diffraction (SAED) pattern matched well with the monoclinic phase of β-Ag_2Te.

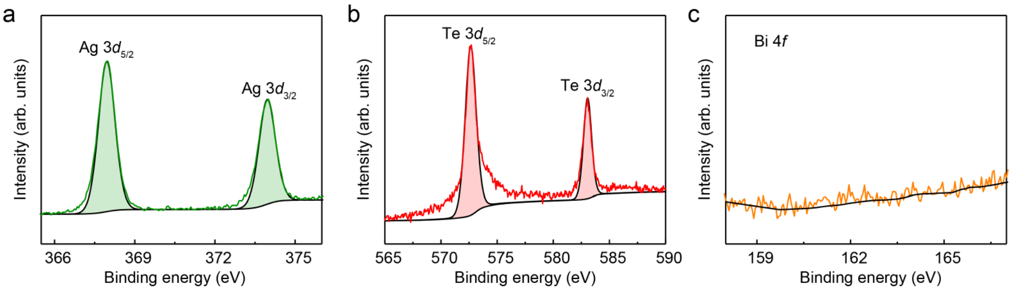

Supplementary Fig. 6 | The XPS analysis of CVD-grown *β*-Ag_2Te on mica. The XPS spectrum for Ag 3*d* **(a)** and Te 3*d* **(b)** can be ascribed to the Ag^{+} and Te^{2-}, which are consistent with the chemical valences in *β*-Ag_2Te. It should be emphasized that no detectable signal of Bi 4*f* **(c)** was obtained, indicating the as-grown nanosheets are pure phase of *β*-Ag_2Te without the Bi dopants, even though Bi_2O_3 powders were involved in the CVD growth process.

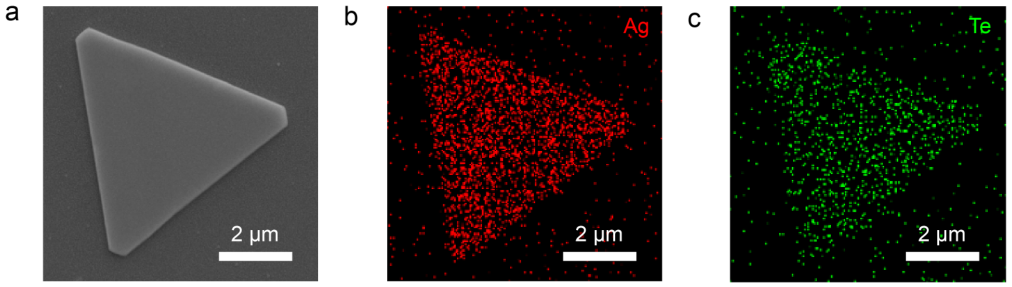

Supplementary Fig. 7 | Quantitative analysis on the elements of *β*-Ag_2Te crystal by SEM EDX mapping. (a-c) SEM image **(a)** and the corresponding element maps for Ag **(b)** and Te **(c)** of an as-synthesized *β*-Ag_2Te nanosheet.

Supplementary Table 1. EDS analysis acquired from 10 different *β*-Ag_2Te nanosheets, showing an averaged atomic ratio of Ag: Te = 2 :1.

Sample	Ag (At%)	Te (At%)	Ag:Te
1	66.7	33.3	2.00
2	66.4	33.6	1.98
3	65.7	34.3	1.92

4	67	33	2.03
5	66.4	33.6	1.98
6	67.3	32.7	2.06
7	67.5	32.5	2.08
8	67.1	32.9	2.04
9	65.6	34.4	1.91
10	67.1	32.9	2.04
Average			2.00

Supplementary Note 2: PHE and AMR measurements on CVD-grown *β*-Ag_2Te nanosheets

We have used a series of experimental tools to analyze the crystalline phase and elemental compositions of as-synthesized samples, including the SEM EDX mapping (averaging the value on 10 samples, Supplementary Fig. 7 and Supplementary Table 1), TEM imaging along two zone axes (Figs. 1h-i, and Supplementary Fig. 5), atomic-resolved EDS mapping (Figs. 1j-k) and XPS measurements (Supplementary Fig. 6). All of them can be assigned to the beta phase of Ag_2Te with a nearly perfect atomic ratio of Ag: Te = ~2 :1. However, we should admit that the possible stoichiometric deviations induced by trace amount of defects, such as 0.001%~0.1%, are almost undetectable by EDX with a detection limit of ~1% and the existing other quantitative tools. Fortunately, trace amount of defects can greatly influence the electrical properties of a material, such as carrier density and conductivity. To this end, one can extract the carrier density of a material by Hall measurement to indirectly reflect the amount of defects in it. As shown in Supplementary Fig. 8, the CVD-grown *β*-Ag_2Te is *n*-type, and has a low carrier density of ~$1.0*10^{18}$ cm^{-2} at room temperature, which is much lower than the value (10^{19}–10^{20} cm^{-3}) of well-known Bi_2Se_3 topological insulator. Let's suppose one defect site donates one electron, thus the defects density for the carrier density of ~10^{18} cm^{-3} can be estimated as 0.01%–0.001%. Such a trace amount of defects are almost impossible to accurately determine by all the existing experimental tools for elemental

analysis.

To investigate the potential microscopic conduction mechanism in β-Ag_2Te, we performed DFT calculations to see the formation energies and dopant type for four possible different defects. As shown in Supplementary Figs. 9a-d, the Te vacancy (V_{Te}) and Ag_{Te} antisite defects act as the electron donor (n-type) in β-Ag_2Te, while the Ag vacancy (V_{Ag}) and Te_{Ag} antisite defects are the acceptor dopants (p-type) instead. That is to say, the system tends to demonstrate *n*-type behavior in Ag-rich environments, while exhibiting p-type characteristics in Ag-poor conditions. This behavior is consistent with the previous experimental results, in which $Ag_{2+x}Te$ and $Ag_{2-x}Te$ are n-type and p-type, respectively[1]. Furthermore, our investigation revealed that the Ag_{Te} substitutional defect has the smallest formation energy (1.175 eV, Supplementary Fig. 9e), thus acting as the preferred *n*-type dopant and matching well the experimental results of Hall measurements (slightly n doped in CVD-grown β-Ag_2Te, Supplementary Fig. 7).

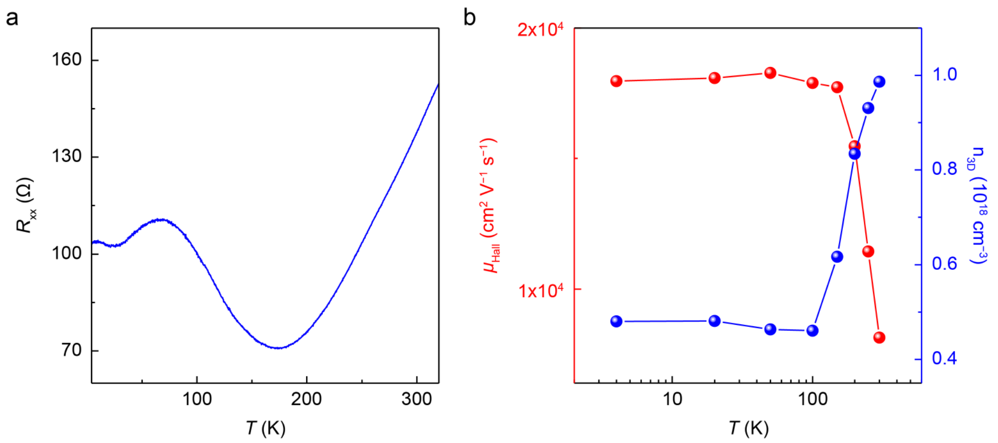

Supplementary Fig. 8 | (a) Longitudinal resistance as a function of temperature ranging from 320 K to 4 K. **(b)** The extracted Hall mobility and carrier density of CVD-grown β-Ag_2Te as a function of temperature measured on the device of Fig. 2 (main text). The room-temperature carrier density can be as low as $1.0*10^{18}$ cm^{-3}, and Hall mobility can be as high as $\sim 8*10^{3}$ cm^2 V^{-1} s^{-1} at room temperature.

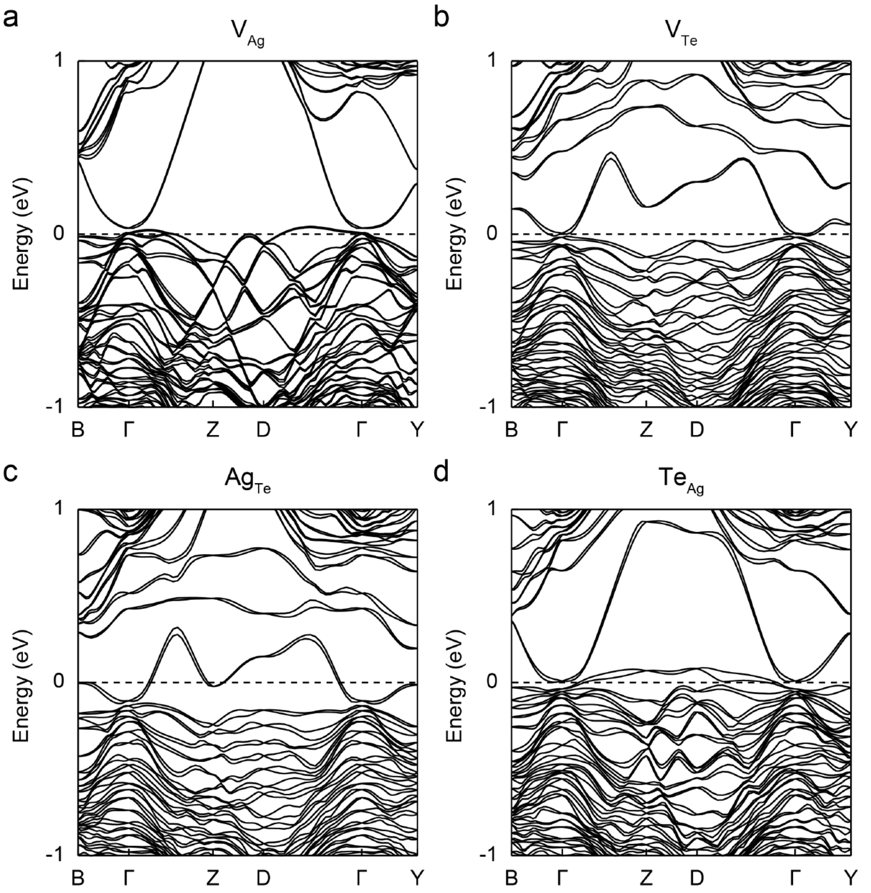

e

Defects	V_{Ag}	V_{Te}	Ag_{Te}	Te_{Ag}
E_f (eV)	2.362	3.529	1.175	1.371

Supplementary Fig. 9 | The band structures (a-d) and formation energies (e) for *β*-Ag_2Te supercell with four possible defects by DFT calculations. Formation energies for four types of defects, encompassing two vacancies (V_{Ag} and V_{Te}) and two substitutional defects (Ag_{Te} and Te_{Ag}), in a 2 × 2 × 2 *β*-Ag_2Te supercell considering SOC. In the case of the Ag_{Te} substitutional defect, one Ag atom substitutes for one Te atom in the supercell. Conversely, for Te_{Ag}, one Te atom substitutes for one Ag atom in the supercell. The formation energy is defined as $E_f = (E_{defect} + E_{atom}) - E_{perfect}$ for vacancy defects and $E_f = (E_{defect} + E_{substituted\ atom} - E_{replacing\ atom}) - E_{perfect}$ for substitutional defects. E_{defect} and $E_{perfect}$ represent the total energy of the supercell with a defect and the perfect supercell, respectively. E_{atom} corresponds to the energy of an individual atom that has been removed to create a vacancy. $E_{substituted\ atom}$ and $E_{replacing\ atom}$ correspond to

the energy of the atom being substituted and the energy of the atom replacing it, respectively.

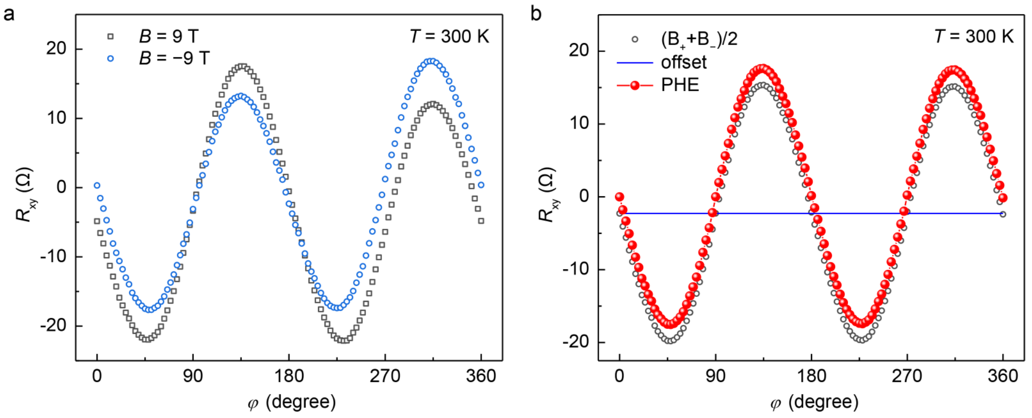

Supplementary Fig. 10 | Data processing of the measured R_{xy}-B results in Figure 2 (main text) to extract the intrinsic PHE data. (a) Raw data of angular-dependent R_{xy} measured in the magnetic field of 9 T (black) and −9 T (blue). **(b)** The corresponding symmetrized result (red) by averaging the measured R_{xy} in both positive and negative field direction. The intrinsic PHE (blue) was obtained by removing a constant offset caused by misalignment.

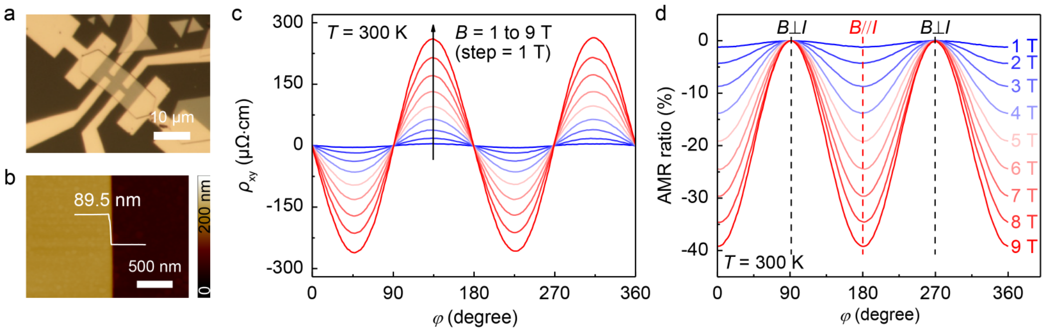

Supplementary Fig. 11 | Another CVD-grown β-Ag_2Te based Hall-bar device with giant room-temperature PHE and AMR ratio. (a) OM image of the as-fabricated Hall-bar device. **(b)** The corresponding AFM image and height profile, showing a thickness of 89.5 nm. **(c)** Angular dependence of planar Hall resistance (R_{xy}) measured at 300 K and various in-plane magnetic fields ranging from 1 to 9 T, showing a giant room-temperature PHE amplitude of ~520 μΩ·cm at 9 T. **(d)** Angular dependence of

AMR ratio measured in different magnetic fields and at 300 K, which can reach a giant value of −39% at 9 T. It is worth noting that Figures c and d were measured immediately after device fabrication.

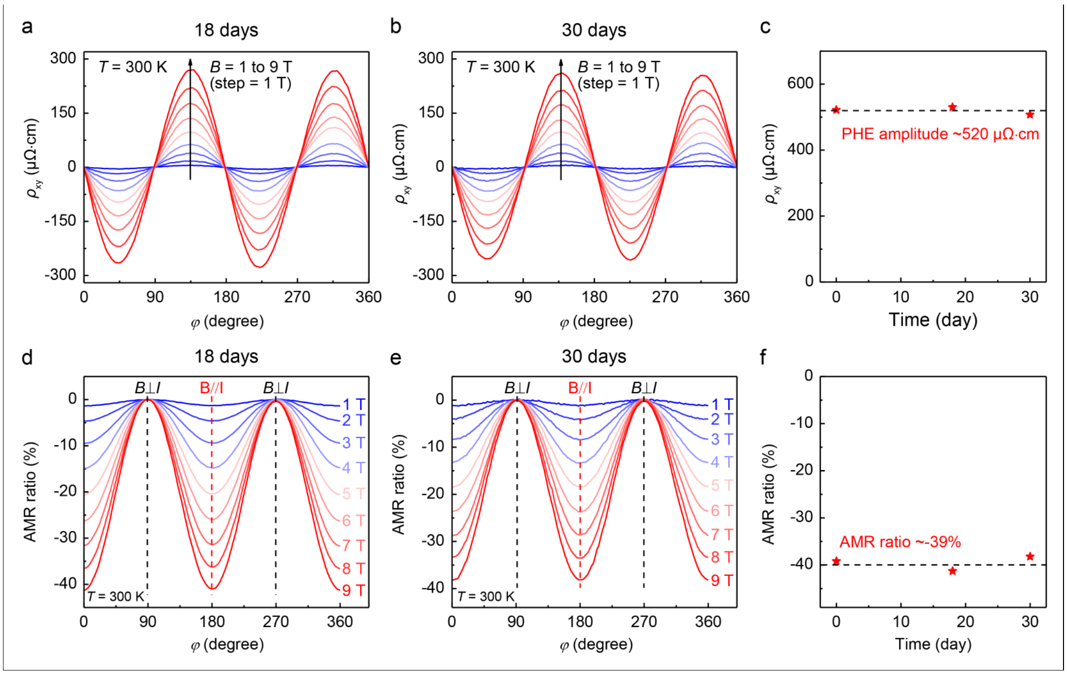

Supplementary Fig. 12 | Evaluating the air stability of the AMR and PHE properties of *β*-Ag_2Te Hall device by multiple Hall measurements on the same device with different air-exposure time. (a, d) Angular dependence of R_{xy} **(a)** and AMR ratio **(d)** measured after exposed to air for 18 days. **(b, e)** Angular dependence of R_{xy} **(b)** and AMR ratio **(e)** measured after exposed to air for 30 days. **(c, f)** Comparison of Angle-dependent R_{xy} **(c)** and ARM ratios **(f)** of the device with different air-exposure time. Only small changes on PHE amplitudes and AMR ratios were observed in *β*-Ag_2Te Hall device even after exposed to air for 1 month.

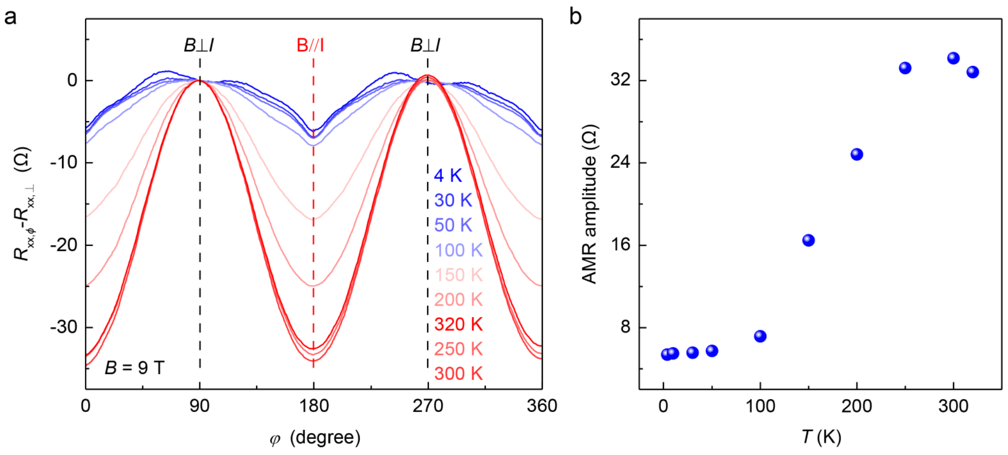

Supplementary Fig. 13 | a Temperature-dependent AMR measurements at varied temperatures from 320 to 4 K. The in-plane magnetic field is kept constant as 9 T. **b** The extracted AMR amplitude as a function of temperature on the basis of **a**. Here, the AMR amplitude is defined as $R_{xx,\perp} - R_{xx,//}$.

Supplementary Table 2. The comparison of AMR ratios and PHE amplitudes between the reported different materials and *β*-Ag_2Te.

Category	Material	AMR ratio	PHE amplitude	Reference
ferromagnet	Fe	0.3% at 300 K	--	[2]
	Co	2% at 300 K	--	[2]
	Ni	2.2% at 300 K	--	[2]
	γ-Fe_4N	−0.5% at 300 K	--	[2]
	$Fe_{20}Ni_{80}$	0.8% at 300 K	--	[3]
	$Cr_{1.2}Te_2$	−0.35% at 300 K	0.6 μΩ·cm at 300 K	[4]
antiferromagnet	Fe_2As	0.04% at 300 K	--	[5]
	$La_{0.7}Sr_{0.3}MnO_3$	0.32% at 300 K	--	[6]
Semimetal	$ZrTe_5$	−7% at 300 K and 14 T	67 μΩ·cm at 200 K and 14 T	[7]
	1-Cd_3As_2	−68% at 2 K and 14 T	40 μΩ·cm at 300 K	[8,9]

	$Co_3Sn_2S_2$	−1% at 100 K	1 μΩ·cm at 100 K and 9 T	10
	$PtSe_2$	−0.05% at 300 K	0.5 μΩ·cm at 300 K	11
	WTe_2	--	0.3 μΩ·cm at 300 K and 14 T	12
	$NiTe_2$	−50% at 2 K and 14 T	0.05 μΩ·cm at 150 K and 14 T	13
Topological insulator	Bi_2Te_3	--	0.25 μΩ·cm at 180 K	14
	Sn-doped $Bi_{1.1}Sb_{0.9}Te_2S$	--	~0 μΩ·cm at 300 K	15
	$Bi_{85}Sb_{15}$	−4% at 200 K	1.2 μΩ·cm at 200 K	16
	β-Ag_2Te	−39% at 300K and 9 T	520 μΩ·cm at 300 K and 9 T	This work

Supplementary Note 3: Theory of planar Hall effect in topological insulators

I. MODEL HAMILTONIAN

We consider energies within the topological insulator bulk band gap where only surface states are relevant. As the surface states are subjected to magnetic field $\mathbf{B}$, the single-particle excitation near the Dirac point can be described by the low-energy effective Hamiltonian[17]

$$\mathcal{H}_{\mathbf{k}} = \frac{\hbar^2 k^2}{2m_{\mathrm{eff}}} + \hbar v_{\mathrm{F}}\left(k_{\mathrm{x}}\sigma_{\mathrm{y}} - k_{\mathrm{y}}\sigma_{\mathrm{x}}\right) - g\mu_{\mathrm{B}}\mathbf{B}\cdot\boldsymbol{\sigma}, \quad (1)$$

where m_{eff} is the effective mass of the band electrons induced by the particle-hole asymmetry, σ represents the spin Pauli matrix and v_{F} denotes the Fermi velocity. g and $\mu_{\mathrm{B}} = e\hbar/(2m_{\mathrm{e}})$ account for the Lande factor and Bohr magneton, respectively,

where m_e is the mass of free electron. By diagonalizing Eq. (1), we can obtain the dispersion $\varepsilon_{\mathbf{k},\eta} = d_{\mathbf{k},0} + \eta|\boldsymbol{d}_{\mathbf{k}}|$ and the corresponding wavefunction

$$\psi_{\mathbf{k},\eta} = \frac{1}{\sqrt{2|\mathbf{d}_{\mathbf{k}}|(|\mathbf{d}_{\mathbf{k}}|-\eta d_{\mathbf{k},z})}} \begin{pmatrix} d_{\mathbf{k},x} - i d_{\mathbf{k},y} \\ \eta|\mathbf{d}_{\mathbf{k}}| - d_{\mathbf{k},z} \end{pmatrix}, \tag{2}$$

in which $d_{\mathbf{k},z} = -g\mu_B B_z, d_{\mathbf{k},x(y)} = \mp\hbar v_F k_{y(x)}$ and $\eta = \pm$ labels the helicity of the Dirac fermions. The in-plane components of the magnetic field will shift the Dirac point away from $\mathbf{k} = 0$ to $\mathbf{k} = \frac{g\mu_B}{\hbar v_F}(B_y, -B_x)$, around which $d_{\mathbf{k},0} = \hbar v_B(k_x \sin\theta_B - k_y \cos\theta_B)$ with $v_B = g\mu_B\sqrt{B_x^2 + B_y^2}/(m_{\text{eff}} v_F)$ and $\theta_B = \tan^{-1}(B_y/B_x)$. As a result, the $d_{\mathbf{k},0}$ term will tilt the Dirac cone, making the Fermi surface deform from a circle to an ellipse. Therefore, the spin structure on the Fermi surface would change with the in-plane magnetic field, leading to the anisotropic electron scattering, as sketched by Fig. 1(a) of the main text.

Here, the tilt direction of the Dirac cone is determined by the magnetic field, so that the electron transport is expected to be sensitive to the magnetic field. For finite temperatures, the electron-phonon interaction is also particularly relevant, as it could make a significant contribution to the transport properties of the surface states. With the electron-phonon coupling taken into accounts, the total Hamiltonian in the language of second quantization can take the form

$$H = \sum_{\mathbf{k}} c_{\mathbf{k}}^\dagger \mathcal{H}_{\mathbf{k}} c_{\mathbf{k}} + \sum_{\mathbf{q}} \hbar\omega_{\mathbf{q}} b_{\mathbf{q}}^\dagger b_{\mathbf{q}} + \sum_{\mathbf{kq}} D_{\mathbf{q}}(b_{\mathbf{q}} + b_{-\mathbf{q}}^\dagger) c_{\mathbf{k+q}}^\dagger c_{\mathbf{k}} + \sum_{\mathbf{kq}} c_{\mathbf{k}}^\dagger U_{\mathbf{q}} c_{\mathbf{k+q}}, \tag{3}$$

where $c_k = (c_{k\uparrow}, c_{k\downarrow})^T$ and b_q are the electron and phonon annihilation operators, respectively. The electron-phonon coupling coefficient reads

$$D_{\mathbf{q}} = -iV_{\mathbf{q}}(\boldsymbol{e}_q \cdot \mathbf{q})\sqrt{\frac{N_{\text{ion}}\hbar}{2M\omega_{\mathbf{q}}}} = -iV_{\mathbf{q}}\sqrt{\frac{N_{\text{ion}}\hbar q}{2Mv_s}}, \tag{4}$$

in which M is the ionic mass, N_{ion} the ionic number and $\omega_{\mathbf{q}} = v_s q$ is the phonon dispersion, with v_s and $\boldsymbol{e}_q$ being respectively the velocity and polarization vector of sound. The last term of Eq. (3) captures the electron-impurity scattering with $U_{\mathbf{q}}$ being the impurity scattering potential.

II. GREEN'S FUNCTIONS AND SELF-ENERGY

The retarded Green's function for the topological surface states is defined as

$$G^{r}_{\mathbf{k},\mathbf{k}'}(t,t')=\frac{1}{i\hbar}\Theta(t-t')\langle\{c_{\mathbf{k}}(t),c^{\dagger}_{\mathbf{k}'}(t')\}\rangle=\langle\langle c_{\mathbf{k}}\,|\,c^{\dagger}_{\mathbf{k}'}\rangle\rangle_{t-t'}. \tag{5}$$

With $\Theta(x)$ being the unit step function. According to the equation of motion

$$\epsilon\langle\langle c_{\mathbf{k}}\,|\,c^{\dagger}_{\mathbf{k}'}\rangle\rangle_{\epsilon}=\langle\{c_{\mathbf{k}},c^{\dagger}_{\mathbf{k}'}\}\rangle+\langle\langle[c_{\mathbf{k}},H]\,|\,c^{\dagger}_{\mathbf{k}'}\rangle\rangle_{\epsilon}, \tag{6}$$

Where $\langle\langle c_{\mathbf{k}}\,|\,c^{\dagger}_{\mathbf{k}'}\rangle\rangle_{\epsilon}$ is the double-time Fourier transform of $\langle\langle c_{\mathbf{k}}\,|\,c^{\dagger}_{\mathbf{k}'}\rangle\rangle_{t-t'}$ we can derive

$$\begin{aligned}\left(\epsilon-\mathcal{H}_{\mathbf{k}}\right)\langle\langle c_{\mathbf{k}}\,|\,c^{\dagger}_{\mathbf{k}'}\rangle\rangle_{\epsilon}&=\delta_{\mathbf{k}\mathbf{k}'}+\sum_{\mathbf{p}}U_{\mathbf{p}}\langle\langle c_{\mathbf{k}+\mathbf{p}}\,|\,c^{\dagger}_{\mathbf{k}'}\rangle\rangle_{\epsilon}\\&+\sum_{\mathbf{q}}D_{\mathbf{q}}\langle\langle b_{\mathbf{q}}c_{\mathbf{k}-\mathbf{q}}\,|\,c^{\dagger}_{\mathbf{k}'}\rangle\rangle_{\epsilon}+\sum_{\mathbf{q}}D_{\mathbf{q}}\langle\langle b^{\dagger}_{\mathbf{q}}c_{\mathbf{k}+\mathbf{q}}\,|\,c^{\dagger}_{\mathbf{k}'}\rangle\rangle_{\epsilon}\end{aligned} \tag{7}$$

with

$$\begin{aligned}\left(\epsilon-\mathcal{H}_{\mathbf{k}+\mathbf{q}}\right)\langle\langle c_{\mathbf{k}+\mathbf{p}}\,|\,c^{\dagger}_{\mathbf{k}'}\rangle\rangle_{\epsilon}&=\delta_{\mathbf{k}+\mathbf{p},\mathbf{k}'}+\sum_{\mathbf{p}}U_{\mathbf{p}}\langle\langle c_{\mathbf{k}+\mathbf{q}+\mathbf{p}}\,|\,c^{\dagger}_{\mathbf{k}'}\rangle\rangle_{\epsilon}\\&+\sum_{\mathbf{p}}D^{\dagger}_{\mathbf{p}}\langle\langle b_{\mathbf{p}}c_{\mathbf{k}+\mathbf{q}-\mathbf{p}}\,|\,c^{\dagger}_{\mathbf{k}'}\rangle\rangle_{\epsilon}+\sum_{\mathbf{p}}D^{\dagger}_{\mathbf{p}}\langle\langle b^{\dagger}_{\mathbf{p}}c_{\mathbf{k}+\mathbf{q}+\mathbf{p}}\,|\,c^{\dagger}_{\mathbf{k}'}\rangle\rangle_{\epsilon},\end{aligned} \tag{8}$$

$$\begin{aligned}\left(\epsilon-\hbar\omega_{\mathbf{q}}-\mathcal{H}_{\mathbf{k}-\mathbf{q}}\right)\langle\langle b_{\mathbf{q}}c_{\mathbf{k}-\mathbf{q}}\,|\,c^{\dagger}_{\mathbf{k}'}\rangle\rangle_{\epsilon}&=\sum_{\mathbf{p}}U_{\mathbf{p}}\langle\langle b_{\mathbf{q}}c_{\mathbf{k}-\mathbf{q}+\mathbf{p}}\,|\,c^{\dagger}_{\mathbf{k}'}\rangle\rangle_{\epsilon}+\sum_{\mathbf{l}}D^{\dagger}_{\mathbf{p}}\langle\langle c_{\mathbf{k}-\mathbf{q}}c^{\dagger}_{\mathbf{l}-\mathbf{q}}c_{\mathbf{l}}\,|\,c^{\dagger}_{\mathbf{k}'}\rangle\rangle_{\epsilon}\\&+\sum_{\mathbf{p}}D^{\dagger}_{\mathbf{p}}\langle\langle c_{\mathbf{k}-\mathbf{q}-\mathbf{p}}b_{\mathbf{q}}b_{\mathbf{p}}\,|\,c^{\dagger}_{\mathbf{k}'}\rangle\rangle_{\epsilon}+\sum_{\mathbf{p}}D^{\dagger}_{\mathbf{p}}\langle\langle c_{\mathbf{k}-\mathbf{q}+\mathbf{p}}b^{\dagger}_{\mathbf{p}}b_{\mathbf{q}}\,|\,c^{\dagger}_{\mathbf{k}'}\rangle\rangle_{\epsilon},\end{aligned} \tag{9}$$

$$\begin{aligned}\left(\epsilon+\hbar\omega_{\mathbf{q}}-\mathcal{H}_{\mathbf{k}+\mathbf{q}}\right)\langle\langle b^{\dagger}_{\mathbf{q}}c_{\mathbf{k}+\mathbf{q}}\,|\,c^{\dagger}_{\mathbf{k}'}\rangle\rangle_{\epsilon}&=\sum_{\mathbf{p}}U_{\mathbf{p}}\langle\langle b^{\dagger}_{\mathbf{q}}c_{\mathbf{k}+\mathbf{q}+\mathbf{p}}\,|\,c^{\dagger}_{\mathbf{k}'}\rangle\rangle_{\epsilon}+\sum_{\mathbf{p}}D^{\dagger}_{\mathbf{p}}\langle\langle c_{\mathbf{k}+\mathbf{q}-\mathbf{p}}b_{\mathbf{p}}b^{\dagger}_{\mathbf{q}}\,|\,c^{\dagger}_{\mathbf{k}'}\rangle\rangle_{\epsilon}\\&-\sum_{\mathbf{l}}D^{\dagger}_{\mathbf{p}}\langle\langle c_{\mathbf{k}+\mathbf{q}}c^{\dagger}_{\mathbf{l}+\mathbf{q}}c_{\mathbf{l}}\,|\,c^{\dagger}_{\mathbf{k}'}\rangle\rangle_{\epsilon}+\sum_{\mathbf{p}}D^{\dagger}_{\mathbf{p}}\langle\langle c_{\mathbf{k}+\mathbf{q}+\mathbf{p}}b^{\dagger}_{\mathbf{q}}b^{\dagger}_{\mathbf{p}}\,|\,c^{\dagger}_{\mathbf{k}'}\rangle\rangle_{\epsilon}.\end{aligned} \tag{10}$$

To close the iterative equation, we perform the mean-field approximation by

contracting the operator pairs, e.g.

$$\langle\langle c_{\mathbf{k-q}} c^{\dagger}_{\mathbf{l-q}} c_{\mathbf{l}} \mid c^{\dagger}_{\mathbf{k}'} \rangle\rangle_{\epsilon} = \langle c_{\mathbf{k-q}} c^{\dagger}_{\mathbf{l-q}} \rangle \langle\langle c_{\mathbf{l}} \mid c^{\dagger}_{\mathbf{k}'} \rangle\rangle_{\epsilon} + \langle c^{\dagger}_{\mathbf{l-q}} c_{\mathbf{l}} \rangle \langle\langle c_{\mathbf{k-q}} \mid c^{\dagger}_{\mathbf{k}'} \rangle\rangle_{\epsilon} - \langle c_{\mathbf{k-q}} c_{\mathbf{l}} \rangle \langle\langle c^{\dagger}_{\mathbf{l-q}} \mid c^{\dagger}_{\mathbf{k}'} \rangle\rangle_{\epsilon},$$

such that we can arrive at

$$\langle\langle c_{\mathbf{k+p}} \mid c^{\dagger}_{\mathbf{k}'} \rangle\rangle_{\epsilon} = \frac{1}{\epsilon - \mathcal{H}_{\mathbf{k+p}}} \delta_{\mathbf{k+p},\mathbf{k}'} + \frac{1}{\epsilon - \mathcal{H}_{\mathbf{k+q}}} \sum_{\mathbf{p}} U_{\mathbf{p}} \langle\langle c_{\mathbf{k+q+p}} \mid c^{\dagger}_{\mathbf{k}'} \rangle\rangle_{\epsilon} \tag{11}$$

$$\begin{aligned} \langle\langle b_{\mathbf{q}} c_{\mathbf{k-q}} \mid c^{\dagger}_{\mathbf{k}'} \rangle\rangle_{\epsilon} = & \frac{1}{\epsilon - \hbar\omega_{\mathbf{q}} - \mathcal{H}_{\mathbf{k-q}}} D^{\dagger}_{\mathbf{q}} \langle b^{\dagger}_{\mathbf{q}} b_{\mathbf{q}} \rangle \langle\langle c_{\mathbf{k}} \mid c^{\dagger}_{\mathbf{k}'} \rangle\rangle_{\epsilon} \\ & + \frac{1}{\epsilon - \hbar\omega_{\mathbf{q}} - \mathcal{H}_{\mathbf{k-q}}} D^{\dagger}_{\mathbf{q}} \langle c_{\mathbf{k-q}} c^{\dagger}_{\mathbf{k-q}} \rangle \langle\langle c_{\mathbf{k}} \mid c^{\dagger}_{\mathbf{k}'} \rangle\rangle_{\epsilon} \end{aligned} \tag{12}$$

$$\begin{aligned} \langle\langle b^{\dagger}_{\mathbf{q}} c_{\mathbf{k+q}} \mid c^{\dagger}_{\mathbf{k}'} \rangle\rangle_{\epsilon} = & \frac{1}{\epsilon + \hbar\omega_{\mathbf{q}} - \mathcal{H}_{\mathbf{k+q}}} D^{\dagger}_{\mathbf{q}} \langle b_{\mathbf{q}} b^{\dagger}_{\mathbf{q}} \rangle \langle\langle c_{\mathbf{k}} \mid c^{\dagger}_{\mathbf{k}'} \rangle\rangle_{\epsilon} \\ & - \frac{1}{\epsilon + \hbar\omega_{\mathbf{q}} - \mathcal{H}_{\mathbf{k+q}}} D^{\dagger}_{\mathbf{q}} \langle c_{\mathbf{k+q}} c^{\dagger}_{\mathbf{k+q}} \rangle \langle\langle c_{\mathbf{k}} \mid c^{\dagger}_{\mathbf{k}'} \rangle\rangle_{\epsilon}. \end{aligned} \tag{13}$$

Finally, we can obtain

$$G^{r}_{\mathbf{k}}(\epsilon) = \langle\langle c_{\mathbf{k}} \mid c^{\dagger}_{\mathbf{k}'} \rangle\rangle_{\epsilon} = g^{r}_{\mathbf{k}}(\epsilon)\left[1 - g^{r}_{\mathbf{k}}(\epsilon)\Sigma_{\mathbf{k}}(\epsilon)\right]^{-1}, \tag{14}$$

in which

$$g^{r}_{\mathbf{k}}(\epsilon) = \sum_{\eta} \frac{|\psi_{\mathbf{k},\eta}\rangle\langle\psi_{\mathbf{k},\eta}|}{\epsilon - \varepsilon_{\mathbf{k},\eta} + i0^{+}} \tag{15}$$

is the unperturbed retarded Green's function for the topological surface states. Within the mean-field approximation, the self-energy can be written as

$$\Sigma_{\mathbf{k}}(\epsilon) = \sum_{\mathbf{q}} \left[U_{\mathbf{q}} g^{r}_{\mathbf{k+q}}(\epsilon) U^{\dagger}_{\mathbf{q}} + \left(n_{\mathbf{q}} - f_{\mathbf{k-q}} + 1\right) D_{\mathbf{q}} g^{r}_{\mathbf{k-q}}\left(\epsilon - \hbar\omega_{\mathbf{q}}\right) D^{\dagger}_{\mathbf{q}} + \left(n_{\mathbf{q}} + f_{\mathbf{k+q}}\right) D_{\mathbf{q}} g^{r}_{\mathbf{k+q}}\left(\epsilon + \hbar\omega_{\mathbf{q}}\right) D^{\dagger}_{\mathbf{q}} \right], \tag{16}$$

where $n_{\mathbf{q}} = \frac{1}{\exp(\hbar\omega_{\mathbf{q}}/k_{\mathrm{B}}T) - 1}$ and $f_{\mathbf{k}} = \frac{1}{\exp[(\varepsilon_{\mathbf{k},\eta} - E_{\mathrm{F}})/k_{\mathrm{B}}T] + 1}$ are the Bose-Einstein and Fermi-Dirac distribution functions, respectively. For a given energy band $\varepsilon_{\mathbf{k},\eta}$, the lifetime broadening function due to the electron scattering is given by $\Gamma_{\mathbf{k},\eta}(\epsilon) = -\mathrm{Im}\left[\langle\psi_{\mathbf{k},\eta}|\Sigma_{\mathbf{k}}(\epsilon)|\psi_{\mathbf{k},\eta}\rangle\right.$, i.e.,

$$\Gamma_{\mathbf{k},\eta} = \pi \sum_{\mathbf{q}} \left|\langle\psi_{\mathbf{k},\eta}|U_{\mathbf{q}}|\psi_{\mathbf{k+q},\eta}\rangle\right|^2 \delta(\epsilon - \varepsilon_{\mathbf{k+q},\eta})$$
$$+\pi \sum_{\mathbf{q}} \frac{1}{\sinh\left(\frac{\hbar\omega_{\mathbf{q}}}{\mathrm{k_B}T}\right)} \left|\langle\psi_{\mathbf{k},\eta}|D_{\mathbf{q}}|\psi_{\mathbf{k-q},\eta}\rangle\right|^2 \delta(\epsilon - \varepsilon_{\mathbf{k-q},\eta} - \hbar\omega_{\mathbf{q}})$$
$$+\pi \sum_{\mathbf{q}} \frac{1}{\sinh\left(\frac{\hbar\omega_{\mathbf{q}}}{\mathrm{k_B}T}\right)} \left|\langle\psi_{\mathbf{k},\eta}|D_{\mathbf{q}}|\psi_{\mathbf{k+q},\eta}\rangle\right|^2 \delta(\epsilon - \varepsilon_{\mathbf{k+q},\eta} + \hbar\omega_{\mathbf{q}}). \quad (17)$$

By substituting Eq. (4) into Eq. (10), we can arrive at

$$\Gamma_{\mathbf{k},\eta} = \pi \sum_{\mathbf{k}'} |U_{\mathbf{k}'-\mathbf{k}}|^2 \cos^2\frac{\phi_k - \phi_k'}{2} \delta(\epsilon - \varepsilon_{\mathbf{k}',\eta})$$
$$+\pi \sum_{\mathbf{k}'} \frac{\mathrm{k_B}T}{2Mv_s^2} \frac{\zeta_q}{\sinh\zeta_q} N_{\mathrm{ion}} |V_{\mathbf{k}'-\mathbf{k}}|^2 \cos^2\frac{\phi_k - \phi_k'}{2} \delta(\epsilon - \varepsilon_{\mathbf{k}',\eta} - \hbar\omega_{\mathbf{q}})$$
$$+\pi \sum_{\mathbf{k}'} \frac{\mathrm{k_B}T}{2Mv_s^2} \frac{\zeta_q}{\sinh\zeta_q} N_{\mathrm{ion}} |V_{\mathbf{k}'-\mathbf{k}}|^2 \cos^2\frac{\phi_k - \phi_k'}{2} \delta(\epsilon - \varepsilon_{\mathbf{k}',\eta} + \hbar\omega_{\mathbf{q}}), \quad (18)$$

where $q = |\mathbf{k}' - \mathbf{k}|$ and $\zeta_q = \hbar\omega_{\mathbf{q}}/\mathrm{k_B}T$, during which we have used the identity

$$\langle\psi_{\mathbf{k},\eta} \mid \psi_{\mathbf{k}',\eta}\rangle = \mathrm{e}^{i\frac{\phi_k - \phi_k'}{2}} \cos\frac{\phi_k - \phi_k'}{2} \quad (19)$$

with $\phi_k = \tan^{-1}(k_y/k_x)$. At relatively high temperatures, i.e., $T \gg T_{\mathrm{BG}}$ with $T_{\mathrm{BG}} = \frac{v_s}{v_F}\frac{2|E_F|}{k_B}$ ($\simeq 4.3$ K for $E_F = 0.1$ eV) being the Bloch-Grüneisen temperature, we can replace $\zeta_q \to \zeta_\epsilon = \frac{v_s}{v_F}\frac{|\epsilon|}{k_B T}$ and reduce the lifetime broadening function as

$$\Gamma_{\mathbf{k},\eta} = \frac{\hbar}{\tau_{\mathrm{im}}} + \frac{\hbar}{\tau_{\mathrm{ion}}} \frac{\mathrm{k_B}T}{2Mv_s^2} \frac{\zeta_\epsilon}{\sinh\zeta_\epsilon} \quad (20)$$

in which

$$\frac{\hbar}{\tau_{\mathrm{im}}} = \pi \sum_{\mathbf{k}'} |U_{\mathbf{k}'-\mathbf{k}}|^2 \cos^2\frac{\phi_{\mathbf{k}} - \phi_{\mathbf{k}}'}{2} \delta(\epsilon - \varepsilon_{\mathbf{k}',\eta}) \quad (21)$$

$$\frac{\hbar}{\tau_{\mathrm{ion}}} = \pi \sum_{k'} N_{\mathrm{ion}} |V_{\mathbf{k}'-\mathbf{k}}|^2 \cos^2\frac{\phi_{\mathbf{k}} - \phi_{\mathbf{k}}'}{2} \left[\delta(\epsilon - \varepsilon_{\mathbf{k}',\eta} - \hbar\omega_{\mathbf{q}}) + \delta(\epsilon - \varepsilon_{\mathbf{k}',\eta} + \hbar\omega_{\mathbf{q}})\right] \quad (22)$$

are temperature-independent, with τ_{im} and τ_{ion} being respectively the effective electron-impurity and electron-ion interaction time. Accordingly, the perturbed retarded

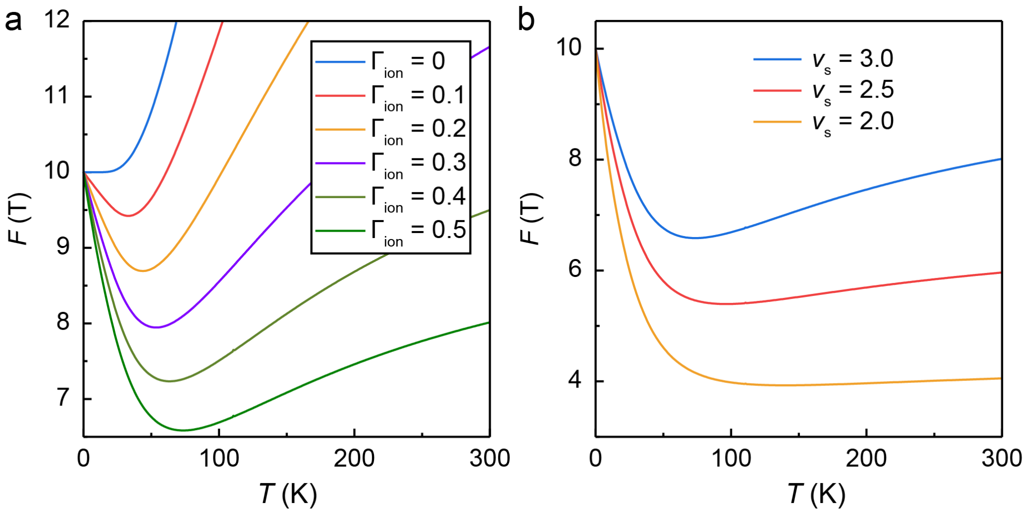

Supplementary Fig. 14 | The temperature factor *F*(*T*). The temperature factor $F(T)$ as a function of temperature for **(a)** $v_s = 3 \times 10^3$ m/s and varied strength of the electron-phonon interaction with $\Gamma_{\text{ion}} = (0, 0.1, 0.2, 0.3, 0.4, 0.5)$ eV, and **(b)** $\Gamma_{\text{ion}} = 0.5$ eV and varied velocity of sound with $v_s = (2.0, 2.5, 3.0) \times 10^3$ m/s. The rest parameters are set as $v_F = 5 \times 10^5\, m/s, \Gamma_0 = 10^{-3}$ eV, $M = 5 \times 10^{-25}$ kg, $m_{\text{eff}} = 0.2\, m_e, B = 1$ Tesla and $g = 10$.

Green's function can be expressed as

$$G_{\mathbf{k}}^r(\epsilon) = \sum_{\eta} \frac{|\psi_{\mathbf{k},\eta}\rangle\langle\psi_{\mathbf{k},\eta}|}{\epsilon - \varepsilon_{\mathbf{k},\eta} + i\Gamma_{\mathbf{k},\eta}}. \tag{23}$$

III. PLANAR HALL EFFECT

The conductivity tensor can be determined from the Kubo-Středa formula[18-21]

$$\sigma_{ij} = -\frac{\hbar e^2}{2\pi S}\int_{-\infty}^{\infty} d\epsilon \sum_{\mathbf{k}} \text{Tr}\{\hat{v}_i[\partial_\epsilon G_{\mathbf{k}}^a(\epsilon)\hat{v}_j G_{\mathbf{k}}^<(\epsilon) - G_{\mathbf{k}}^<(\epsilon)\hat{v}_j\, \partial_\epsilon G_{\mathbf{k}}^r(\epsilon)]\}, \tag{24}$$

in which $\hat{v}_j = \partial\mathcal{H}_{\mathbf{k}}/(\hbar\,\partial k_j)$ is the group velocity operator for the surface states, S denotes the sample area and $G_{\mathbf{k}}^<(\epsilon) = [G_{\mathbf{k}}^a(\epsilon) - G_{\mathbf{k}}^r(\epsilon)]f(\epsilon)$ is the less Green's function, with $G_{\mathbf{k}}^a(\epsilon) = [G_{\mathbf{k}}^r(\epsilon)]^\dagger$ being the advanced Green's function. By substituting Eq. (16) into Eq. (17), we can derive the conductivity tensor as

$$\sigma_{\rm ij} = -\frac{2\hbar e^2}{\pi S}\int_{-\infty}^{\infty} d\epsilon f(\epsilon)\,{\rm Im}\sum_{\mathbf{k},\eta\eta'} \frac{\langle\psi_{\mathbf{k},\eta}|\hat{v}_{\rm i}|\psi_{\mathbf{k},\eta'}\rangle\langle\psi_{\mathbf{k},\eta'}|\hat{v}_{\rm j}|\psi_{\mathbf{k},\eta}\rangle}{\left(\epsilon-\varepsilon_{\mathbf{k},\eta}+i\Gamma_{\mathbf{k},\eta}\right)^2}\frac{\Gamma_{\mathbf{k},\eta'}}{\left(\epsilon-\varepsilon_{\mathbf{k},\eta'}\right)^2+\Gamma_{\mathbf{k},\eta'}^2} \tag{25}$$

In terms of $k_\parallel = k\cos(\phi_k-\theta_{\rm B})$ and $k_\perp = k\sin(\phi_k-\theta_{\rm B})$, the dispersion reads $\varepsilon_{\mathbf{k},\eta} = -\hbar v_{\rm B}k_\perp + \eta\hbar v_{\rm F}\sqrt{k_\parallel^2+k_\perp^2}$. As the in-plane magnetic field can not gap the spectrum, the interband contribution can be neglected, such that the conductivity can be rewritten as

$$\sigma_{\rm ij} = \sigma_\parallel\frac{\partial k_\parallel}{\partial k_{\rm i}}\frac{\partial k_\parallel}{\partial k_{\rm j}} + \sigma_\perp\frac{\partial k_\perp}{\partial k_{\rm i}}\frac{\partial k_\perp}{\partial k_{\rm j}} + \sigma_{\parallel,\perp}\left(\frac{\partial k_\parallel}{\partial k_{\rm i}}\frac{\partial k_\perp}{\partial k_{\rm j}}+\frac{\partial k_\perp}{\partial k_{\rm i}}\frac{\partial k_\parallel}{\partial k_{\rm j}}\right). \tag{26}$$

Here, we refer to $\sigma_\parallel = \sigma_{\parallel,\parallel}$ and $\sigma_\perp = \sigma_{\perp,\perp}$, where

$$\sigma_{\alpha,\beta} = -\int_{-\infty}^{\infty} d\epsilon\,\partial_\epsilon f(\epsilon)\sigma_{\alpha,\beta}(\epsilon) \tag{27}$$

with $\alpha,\beta = \parallel,\perp$ and

$$\sigma_{\alpha,\beta}(\epsilon) = \frac{e^2}{h}\frac{1}{2\pi^2}\sum_\eta\int_{-\infty}^{\infty}dk_\parallel\int_{-\infty}^{\infty}dk_\perp\frac{\partial\varepsilon_{\mathbf{k},\eta}}{\partial k_\alpha}\frac{\partial\varepsilon_{\mathbf{k},\eta}}{\partial k_\beta}\left[\frac{\Gamma_{\mathbf{k},\eta}}{\left(\epsilon-\varepsilon_{\mathbf{k},\eta}\right)^2+\Gamma_{\mathbf{k},\eta}^2}\right]^2. \tag{28}$$

As a result,

$$\sigma_{\rm xy}(\epsilon) = [\sigma_\parallel(\epsilon)-\sigma_\perp(\epsilon)]\sin\theta_{\rm B}\cos\theta_{\rm B} + \sigma_{\parallel,\perp}(\epsilon)\cos 2\theta_{\rm B} \tag{29}$$

and the Hall resistivity is given by

$$\rho_{\rm xy} = -\frac{\sigma_{\rm xy}}{\sigma_{\rm xx}\sigma_{\rm yy}-\sigma_{\rm xy}\sigma_{\rm yx}} = \frac{\sigma_\perp-\sigma_\parallel}{\sigma_\parallel\sigma_\perp-\sigma_{\parallel,\perp}^2}\sin\theta_{\rm B}\cos\theta_{\rm B} - \frac{\sigma_{\parallel,\perp}}{\sigma_\parallel\sigma_\perp-\sigma_{\parallel,\perp}^2}\cos 2\theta_{\rm B}. \tag{30}$$

To leading order in the magnetic field, we find $\sigma_{\parallel,\perp}$ at least $\propto O(\lambda_{\rm B}^4)$, which is negligible small and

$$\sigma_\parallel = \frac{e^2}{h}\frac{1-\sqrt{1-\lambda_{\rm B}^2}}{\lambda_{\rm B}^2\sqrt{1-\lambda_{\rm B}^2}}\mathcal{F}(T), \tag{31}$$

$$\sigma_\perp = \frac{e^2}{h}\frac{1-\sqrt{1-\lambda_{\rm B}^2}}{\lambda_{\rm B}^2}\mathcal{F}(T), \tag{32}$$

where the temperature factor is given by

$$F(T) = -\int_{-\infty}^{\infty} d\epsilon\,\partial_\epsilon f(\epsilon)\frac{|\epsilon|}{\Gamma(\epsilon)} \tag{33}$$

with

$$\Gamma(\epsilon) = \Gamma_0 + \frac{|\epsilon|}{2Mv_F^2}\frac{1}{\lambda_s \sinh\frac{\lambda_s|\epsilon|}{k_B T}}\Gamma_{ion}. \tag{34}$$

Here, we noted $\lambda_s = v_s/v_F, \lambda_B = v_B/v_F, \Gamma_0 = \hbar/\tau_{im}$ and $\Gamma_{ion} = \hbar/\tau_{ion}$ for brevity. Finally, the Hall resistivity takes the form

$$\rho_{xy} = \left(\frac{1}{\sigma_\parallel} - \frac{1}{\sigma_\perp}\right)\sin\theta_B\cos\theta_B = -\frac{h}{e^2}\frac{\lambda_B^2}{\mathcal{F}(T)}\sin\theta_B\cos\theta_B. \tag{35}$$

The amplitude of the Hall resistivity can be determined as

$$\Delta\rho = \frac{h}{e^2}\frac{\lambda_B^2}{\mathcal{F}(T)} = \frac{1}{\mathcal{F}(T)}\frac{m_e^2}{m_{eff}^2}B^2\rho_0, \tag{36}$$

where B is dimensionless when we choose

$$\rho_0 = \frac{h}{e^2}\frac{g^2\mu_B^2[B = 1\text{ Tesla}]^2}{m_e^2 v_F^4} \tag{37}$$

as the unit of resistivity. With the constants $\hbar = 6.58 \times 10^{-16}$ eV $\cdot$ s, $m_e = 9.1 \times 10^{-31}$ kg and $\mu_B = 5.79 \times 10^{-5}$ eV/Tesla, together with the Fermi velocity $v_F = 5 \times 10^5$ m/s, we can evaluate $\rho_0 = 0.43$ mΩ for $g = 10$. Also, for the parameters $\tau_{im} \sim 10^{-13}$ s, $\tau_{ion} \sim 10^{-15}$ s and $M \sim 10^{-25}$ kg, we can estimate $\Gamma_0 \sim 10^{-3}$ eV, $\Gamma_{ion} \sim 10^{-1}$ eV and $Mv_F^2 \sim 10^4$ eV.

The evolution of the temperature factor $F(T)$ with the parameters is plotted in Supplementary Fig. 14. As can be seen, the temperature factor develops a valley at $T = T_p$, which corresponds to a peak in the resistivity, as displayed in Fig.3 of the main text. The minimum value of $F(T)$ decreases with either increasing the electron-phonon interaction or decreasing the sound velocity. At the same time, the value of T_p will increase, making the giant room-temperature PHE realizable.

The unconventional non-monotonic temperature of PHE amplitude can be obtained based on our model, which fits well with the one observed in β-Ag_2Te and other topological insulators. Moreover, with decreasing the sound velocities and effective mass, the peak temperature (T_p) gradually moves to a higher temperature,

accompanied by a significantly enhanced PHE amplitude (Supplementary Fig. 15). It is worth noting that our model is a qualitative analysis, rather than a quantitative tool, to see how the PHE amplitude changes with the parameters and thus help us to understand and figure out the possible origin of large room-temperature AMR ratio and PHE in β-Ag_2Te system.

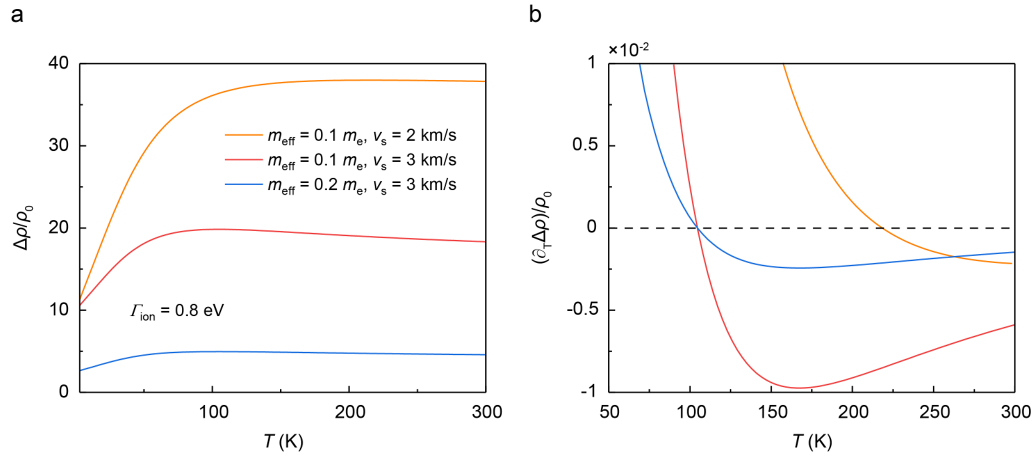

Supplementary Fig. 15 | (a) The PHE amplitude $\Delta\rho/\rho_0$ vs temperature for varied sound velocities (v_s) and effective masses (m_{eff}). **(b)** The differential form of $\Delta\rho/\rho_0$ as a function of temperature, indicating that the peak temperature (T_p) gradually moves to a higher temperature with decreasing the v_s.

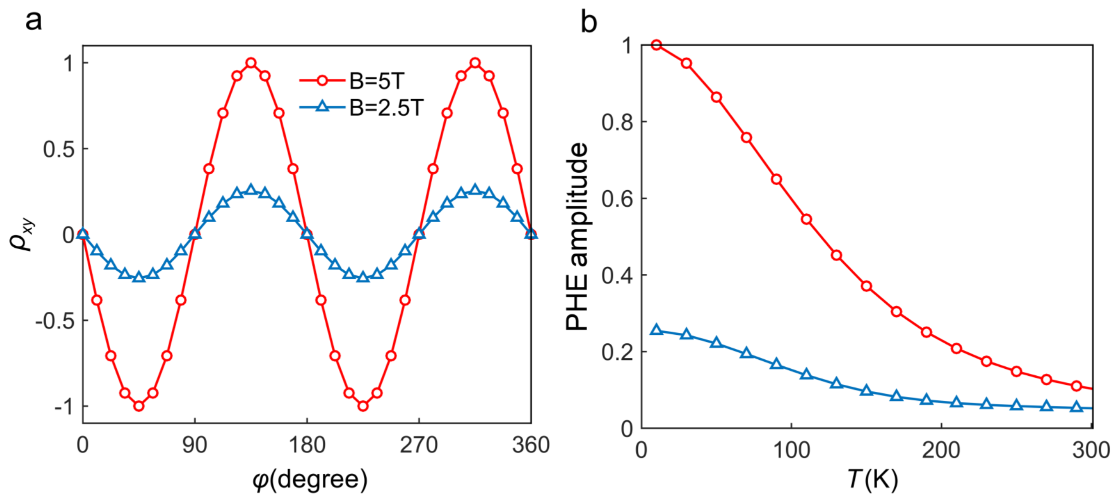

Supplementary Fig. 16 | The PHE contributed from the bulk state of 3D TI s. (a)

Angular dependence of the Hall resistivity at T=30K. **(b)** The PHE amplitude as a function of temperature. The resistivity is normalized by its maximum value in both figures and the Fermi energy is set as 0.23 meV above the bottom of the conduction bands.

We also evaluate the contribution of the bulk state to the PHE, based on the well-accepted bulk Hamiltonian of 3D TI [22,23]. The angular and temperature dependences of the PHE induced by the bulk states are plotted in Supplementary Fig. 16., showing a traditional sin2φ angle dependence. However, with temperature increasing, the PHE amplitude will monotonically decrease, which means that the amplitude of the PHE is higher at low temperatures and smaller at room temperature. Obviously, this is completely opposite to the experimental phenomenon in Fig. 2e of the main text. Therefore, the contribution from the bulk state is not the essential reason for the arising giant room-temperature PHE, and the electron-phonon scattering on the surface states mentioned above may be the dominant cause.

Supplementary Note 4: Fabrication and performance of β-Ag_2Te-based AMR sensor

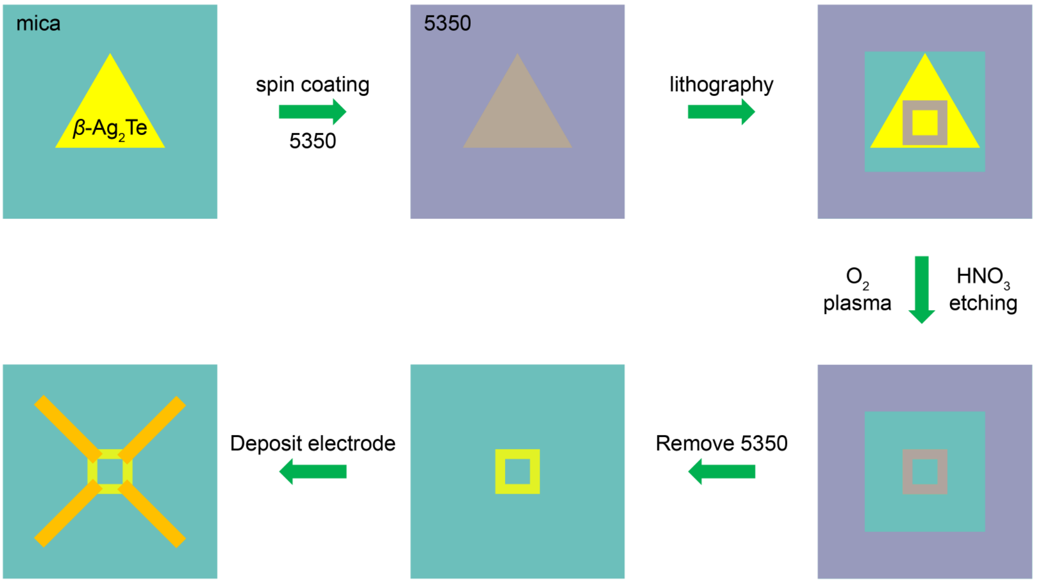

Supplementary Fig. 17 | Schematic illustration of the process to fabricate the AMR sensor with a Wheatstone bridge configuration based on one β-Ag_2Te nanoplate.

Typically, it involves the following steps: 1) using the maskless laser direct writing system (Microlab III) to pattern the AR-P-5350 mask on β-Ag_2Te; 2) immersing the AR-P-5350/β-Ag_2Te/mica after exposed in concentrated HNO_3 solution (~34%, 7.6 mol/L) for about one minute; 3) removing the AR-P-5350 with hot acetone; 4) using another EBL step and thermal evaporation to deposit the contact electrodes.

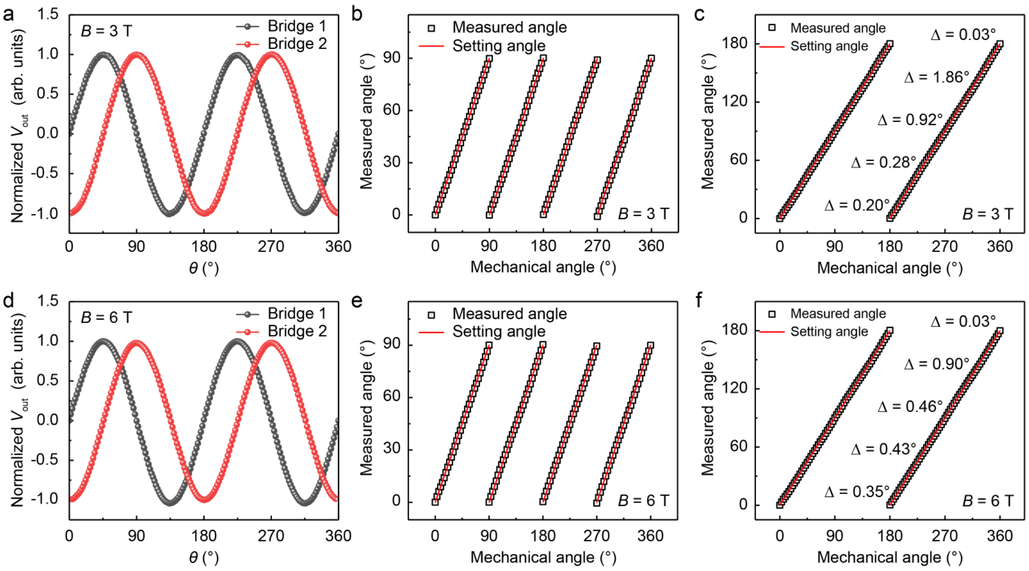

Supplementary Fig. 18 | The angular error analysis of the AMR sensor (Figure 4, main text) at various magnetic fields. (a-c) B = 3 T. **(d-f)** B = 6 T, showing a small angular error.

Supplementary References

1 Schnyders, H. S., Saboungi, M. L. & Rosenbaum, T. F. Magnetoresistance in n- and p-type Ag_2Te: Mechanisms and applications. *Appl. Phys. Lett.* **76**, 1710-1712 (2000).

2 Kokado, S., Tsunoda, M., Harigaya, K. & Sakuma, A. Anisotropic magnetoresistance effects in Fe, Co, Ni, Fe_4N, and half-metallic ferromagnet: A systematic analysis. *J. Phys. Soc. Jpn.* **81**, 024705 (2012).

3 Zhang, W., Wu, T., Peng, B. & Zhang, W. Resistivity dependence of the spin mixing conductance and the anisotropic magnetoresistance in permalloy. *J. Alloys Compd.* **696**, 234-238 (2017).

4 Ma, X. *et al.* Anisotropic magnetoresistance and planar Hall effect in layered room-temperature ferromagnet $Cr_{1.2}Te_2$. *ACS Appl. Electron. Mater.* **5**, 2838-2844 (2023).

5 Wu, J., Karigerasi, M. H., Shoemaker, D. P., Lorenz, V. O. & Cahill, D. G. Temperature dependence of the anisotropic magnetoresistance of the metallic antiferromagnet Fe_2As. *Phys. Rev. A* **15**, 054038 (2021).

6 Perna, P. *et al.* Engineering large anisotropic magnetoresistance in $La_{0.7}Sr_{0.3}MnO_3$ films at room temperature. *Adv. Funct. Mater.* **27**, 1700664 (2017).

7 Li, P., Zhang, C. H., Zhang, J. W., Wen, Y. & Zhang, X. X. Giant planar Hall effect in the Dirac semimetal $ZrTe_{5-\delta}$. *Phys. Rev. B* **98**, 121108 (2018).

8 Li, H., Wang, H.-W., He, H., Wang, J. & Shen, S.-Q. Giant anisotropic magnetoresistance and planar Hall effect in the Dirac semimetal Cd_3As_2. *Phys. Rev. B* **97**, 201110 (2018).

9 Wu, M. *et al.* Probing the chiral anomaly by planar Hall effect in Dirac semimetal Cd_3As_2 nanoplates. *Phys. Rev. B* **98**, 161110 (2018).

10 Yang, S.-Y. *et al.* Field-modulated anomalous Hall conductivity and planar Hall effect in $Co_3Sn_2S_2$ nanoflakes. *Nano Lett.* **20**, 7860-7867 (2020).

11 Li, Z. *et al.* Planar Hall effect in $PtSe_2$. *J. Appl. Phys.* **127**, 054306 (2020).

12 Li, P. *et al.* Anisotropic planar Hall effect in the type-II topological Weyl semimetal WTe_2. *Phys. Rev. B* **100**, 205128 (2019).

13 Liu, Q. *et al.* Nontopological origin of the planar Hall effect in the type-II Dirac semimetal $NiTe_2$. *Phys. Rev. B* **99**, 155119 (2019).

14 Bhardwaj, A., Prasad P, S., Raman, K. V. & Suri, D. Observation of planar Hall effect in topological insulator—Bi_2Te_3. *Appl. Phys. Lett.* **118,** 241901 (2021).

15 Wu, B. *et al.* Oscillating planar Hall response in bulk crystal of topological insulator Sn doped $Bi_{1.1}Sb_{0.9}Te_2S$. *Appl. Phys. Lett.* **113**, 011902 (2018).

16 Budhani, R. C., Higgins, J. S., McAlmont, D. & Paglione, J. Planar Hall effect in c-axis textured films of $Bi_{85}Sb_{15}$ topological insulator. *AIP Adv.* **11**, 055020

(2021).

17 Zheng, S. *et al.* Origin of planar Hall effect on the surface of topological insulators: Tilt of Dirac cone by an in-plane magnetic field. *Phys. Rev. B* **101**, 041408 (2020).

18 Deng, M. *et al.* Competing effects of magnetic impurities in the anomalous Hall effect on the surface of a topological insulator. *Phys. Rev. B* **94**, 235116 (2016).

19 Sinitsyn, N. A., Hill, J. E., Min, H., Sinova, J. & MacDonald, A. H. Charge and spin Hall conductivity in metallic graphene. *Phys. Rev. Lett.* **97**, 106804 (2006).

20 Yang, Y. *et al.* Anomalous Nernst effect on a magnetically doped topological insulator surface: A Green's function approach. *Phys. Rev. B* **98**, 235152 (2018).

21 Weng, Z. *et al.* Magnon scattering modulated planar Hall effect in a ferromagnet/topological insulator heterostructure. *Phys. Rev. B* **106**, 195134 (2022).

22 Dai, X., Du, Z. Z. & Lu, H.-Z. Negative magnetoresistance without chiral anomaly in topological insulators. *Phys. Rev. Lett.* **119**, 166601 (2017).

23 Nandy, S., Taraphder, A. & Tewari, S. Berry phase theory of planar Hall effect in topological insulators. *Sci. Rep.* **8**, 14983 (2018).
